# Diagnostic Performance of a Novel AI–Guided Coronary Computed Tomography Algorithm for Predicting Myocardial Ischemia (AI-QCT_ISCHEMIA_) Across Sex and Age Subgroups

**DOI:** 10.1016/j.jscai.2025.104064

**Published:** 2025-12-30

**Authors:** Putri Annisa Kamila, Tara Hojjati, Nick S. Nurmohamed, Ibrahim Danad, Yipu Ding, Ruurt A. Jukema, Pieter G. Raijmakers, Roel S. Driessen, Michiel J. Bom, Pepijn van Diemen, Gianluca Pontone, Daniele Andreini, Hyuk-Jae Chang, Richard J. Katz, Andrew D. Choi, Paul Knaapen, Jeroen J. Bax, Alexander van Rosendael, CREDENCE and PACIFIC-1 Investigators, Ran Heo, Hyung-Bok Park, Hugo Marques, Wijnand J. Stuijfzand, Jung Hyun Choi, Joon-Hyung Doh, Ae-Young Her, Bon-Kwon Koo, Chang-Wook Nam, Sang-Hoon Shin, Jason Cole, Alessia Gimelli, Muhammad Akram Khan, Bin Lu, Yang Gao, Faisal Nabi, Mouaz H. Al-Mallah, Ryo Nakazato, U. Joseph Schoepf, Randall C. Thompson, James J. Jang, Michael Ridner, Chris Rowan, Erick Avelar, Philippe Généreux, Guus A. de Waard

**Affiliations:** 1 Division of Cardiology, Department of Internal Medicine, Hanyang University Medical Center, Seoul, Korea; 2 Division of Cardiology, Department of Internal Medicine, International St. Mary's Hospital, Catholic Kwandong University College of Medicine, Incheon, South Korea; 3 Faculdade de Medicina da Universidade Católica Portuguesa, Lisboa, Portugal; 4 Department of Cardiology, Amsterdam UMC, Vrije Universiteit Amsterdam, Amsterdam, The Netherlands; 5 Department of Cardiology, Pusan National University Hospital, Busan, South Korea; 6 Division of Cardiology, Inje University Ilsan Paik Hospital, South Korea; 7 Division of Cardiology, Department of Internal Medicine, Kangwon National University, College of Medicine, Kangwon National University School of Medicine, Chuncheon, South Korea; 8 Department of Internal Medicine, Seoul National University Hospital, Seoul, South Korea; 9 Cardiovascular Center, Keimyung University Dongsan Hospital, Daegu, South Korea; 10 Division of Cardiology, Department of Internal Medicine, Ewha Women's University Seoul Hospital, Seoul, South Korea; 11 Mobile Cardiology Associates, Mobile, Alabama, USA; 12 Department of Imaging, Fondazione Toscana Gabriele Monasterio, Pisa, Italy; 13 Cardiac Center of Texas, McKinney, Texas, USA; 14 State Key Laboratory of Cardiovascular Disease, Fuwai Hospital, Beijing, China; 15 Houston Methodist Hospital, Houston, Texas, USA; 16 Cardiovascular Center, St. Luke's International Hospital, Tokyo, Japan; 17 Medical University of South Carolina, Charleston, South Carolina, USA; 18 Saint Luke's Mid America Heart Institute, Kansas, Missouri, USA; 19 San Jose Medical Center, Kaiser Permanente, San Jose, California, USA; 20 University of Alabama-Birmingham, Birmingham, Alabama, USA; 21 University of Wisconsin-Madison, Madison, Wisconsin, USA; 22 Oconee Heart and Vascular Center, University of GA School of Medicine, Athens, Georgia, USA; 23 Morristown Medical Center, Morristown, New Jersey, USA; a Department of Cardiology, Leiden University Medical Center, Leiden, the Netherlands; b Faculty of Medicine, Universitas Brawijaya, Malang, Indonesia; c The College of Liberal Arts and Sciences, Arizona State University, Tempe, Arizona; d Department of Cardiology, Amsterdam UMC, Vrije Universiteit Amsterdam, Amsterdam, the Netherlands; e Department of Vascular Medicine, Amsterdam UMC, University of Amsterdam, Amsterdam, the Netherlands; f Department of Cardiology, Radboud University Medical Center, Nijmegen, the Netherlands; g School of Medicine, Nankai University, Tianjin, China; h Department of Radiology and Nuclear Medicine, Amsterdam UMC, Vrije Universiteit Amsterdam, Amsterdam, the Netherlands; i Department of Cardiovascular Imaging, Centro Cardiologico Monzino, IRCCS, Milan, Italy; j Department of Biomedical, Surgical and Dental Sciences, University of Milan, Milan, Italy; k Department of University Cardiology and Cardiac Imaging, IRCCS Ospedale Galeazzi Sant'Ambrogio, Milan, Italy; l Division of Cardiology, Severance Cardiovascular Hospital and Severance Biomedical Science Institute, Yonsei University College of Medicine, Yonsei University Health System, Seoul, South Korea; m Division of Cardiology, The George Washington University School of Medicine, Washington, District of Columbia; n Turku University Hospital and University of Turku, Turku, Finland

**Keywords:** artificial intelligence, coronary computed tomography angiography, coronary artery disease

## Abstract

**Background:**

AI-QCT_ISCHEMIA_ is a novel artificial intelligence algorithm that predicts myocardial ischemia using quantitative features from coronary computed tomography angiography, providing a noninvasive alternative to functional imaging. However, its diagnostic performance across key demographic subgroups, particularly by sex and age, remains underexplored. We aimed to evaluate the diagnostic performance of AI-QCT_ISCHEMIA_ for predicting myocardial ischemia across these subgroups.

**Methods:**

This post-hoc analysis included symptomatic patients with suspected coronary artery disease from the CREDENCE (Computed Tomographic Evaluation of Atherosclerotic Determinants of Myocardial Ischemia) (n = 305; 868 vessels) and PACIFIC-1 (Comparison of Coronary Computed Tomography Angiography, Single Photon Emission Computed Tomography [SPECT], Positron Emission Tomography [PET], and Hybrid Imaging for Diagnosis of Ischemic Heart Disease Determined by Fractional Flow Reserve) (n = 208; 612 vessels) studies. All patients underwent coronary computed tomography angiography, myocardial perfusion imaging (SPECT and/or PET), and invasive coronary angiography with 3-vessel fractional flow reserve as the reference standard. Diagnostic performance was evaluated at the vessel level using receiver operating characteristic analysis and under the curve (AUC), stratified by sex and age groups.

**Results:**

In computed tomographic evaluation of atherosclerotic determinants of myocardial ischemia, AI-QCT_ISCHEMIA_ demonstrated higher diagnostic performance than myocardial perfusion imaging, with AUCs of 0.87 vs 0.63 in men and 0.85 vs 0.71 in women (*P* < .001 for both). Similarly, in older (≥65 years) and younger (<65 years) patients, AUCs were 0.85 vs 0.67 and 0.87 vs 0.63 (*P* < .001 for both). In PACIFIC-1, AI-QCT_ISCHEMIA_ outperformed SPECT in men (AUC = 0.86 vs 0.67; *P* < .001) and women (0.81 vs 0.65; *P* < .001) while performing comparably with PET (0.86 vs 0.82; *P* = .140; 0.81 vs 0.72; *P* = .214). In older patients, AI-QCT_ISCHEMIA_ showed higher performance than SPECT (0.85 vs 0.73; *P* < .001) and was similar to PET (0.85 vs 0.86; *P* = .816). In younger patients, it also outperformed SPECT (0.87 vs 0.66; *P* < .001) with comparable performance with PET (0.87 vs 0.84; *P* = .338).

**Conclusions:**

AI-QCT_ISCHEMIA_ demonstrated consistently high diagnostic performance to detect myocardial ischemia across sex and age groups, significantly outperforming SPECT and showing comparable performance with PET, supporting its role as a noninvasive alternative for ischemia assessment.

## Introduction

Personalized strategies for diagnosing coronary artery disease (CAD) have become increasingly important as clinical evidence highlights heterogeneity in disease presentation across populations.^1^ Traditionally, myocardial perfusion imaging (MPI) with single-photon emission computed tomography (SPECT) or positron emission tomography (PET) has been the standard for functional assessment; however, these approaches are limited by the lack of anatomical assessment and substantial health care costs.^2^^,^^3^ In contrast, coronary computed tomography angiography (CCTA) offers direct visualization of coronary artery anatomy and has achieved a class 1 recommendation as a first-line diagnostic test for suspected CAD, reflecting its growing clinical importance.^4^^,^^5^

The integration of artificial intelligence (AI) in CCTA analysis has further enhanced its diagnostic potential. A novel approach, AI-QCT_ISCHEMIA_ (Cleerly, Inc), leverages AI-driven, quantitative analysis from CCTA to predict myocardial ischemia. Prior studies have demonstrated that AI-QCT_ISCHEMIA_ has a high diagnostic accuracy for the detection of reduced fractional flow reserve (FFR), surpassing SPECT.^6^^,^^7^ Furthermore, the algorithm has shown strong prognostic value for predicting major adverse cardiovascular events (MACE).^8^

Despite these advancements, the sex- and age-specific diagnostic performance of AI-QCT_ISCHEMIA_ remains insufficiently characterized. Given established differences in the pathophysiology and clinical presentation of CAD between sexes and across age groups,^9^ it is essential to evaluate diagnostic tools within these demographic contexts. This study evaluates the ability of AI-QCT_ISCHEMIA_ to predict ischemia in sex- and age-stratified cohorts, addressing a critical evidence gap and advancing personalized cardiovascular diagnostic assessments.

## Materials and methods

### Study population

This post-hoc analysis was conducted using data from the computed tomographic evaluation of atherosclerotic determinants of myocardial ischemia (CREDENCE) (NCT02173275) and comparison of CCTA, SPECT, PET, and hybrid imaging for diagnosis of ischemic heart disease determined by FFR (PACIFIC-1) (NCT01521468) trials^10^^,^^11^ to assess the accuracy of AI-QCT_ISCHEMIA_ across various demographic subgroups. The CREDENCE trial was a prospective, multicenter study that enrolled 305 patients (868 vessels) from the validation cohort, with stable symptoms without prior diagnosis of CAD. Participants were recruited from 23 centers between 2014 and 2017. The PACIFIC-1 trial, conducted from 2012 to 2014, included 208 patients (612 vessels) with new-onset, stable chest pain and suspected CAD. All participants in both trials underwent invasive coronary angiography (ICA) with 3-vessel FFR measurements, the reference standard for diagnosing ischemia. In addition to ICA and FFR, diagnostic assessments included CCTA and MPI using SPECT and/or PET. Both studies were approved by the local ethics committees, and all participants provided written informed consent. The studies were conducted in accordance with the Declaration of Helsinki.

### CCTA acquisition

In both studies, CCTA was performed using single- or dual-source CT scanners with at least 64-detector rows. All images were acquired in accordance with the guidelines provided by the Society of Cardiovascular Computed Tomography.^12^

### AI-QCT_ISCHEMIA_ derived from coronary CTA

The CCTA scans from both studies were analyzed using the Food and Drug Administration–cleared AI-QCT algorithm.^13^ This software employs convolutional neural networks, including 3-dimensional U-Net and Visual Geometry Group (VGG) network variants, as described previously.^14^ The algorithm performs multiple functions: it assesses image quality, segments and labels coronary arteries, evaluates blood vessel walls, determines vessel contours, and characterizes plaque. The AI-QCT algorithm generates several key quantitative metrics, including stenosis parameters; plaque measures such as total plaque volume, composition-specific volumes, and plaque burden; and vascular morphology, including vessel length, lumen and vessel volume, and average lumen area. It also evaluates the diffuseness of atherosclerosis by summing plaque volumes and lengths across all affected segments.

A predictive model for ischemia detection, AI-QCT_ISCHEMIA_, was developed using quantitative parameters derived from the AI-QCT algorithm, based on the CREDENCE derivation cohort comprising 307 patients, as described in a previous study.^7^ This model identifies vessel-specific ischemia in a binary manner, defined by an invasive FFR ≤0.80. Model construction was based on AI-QCT measurements and invasive FFR data, with investigators blinded to all clinical, diagnostic, and outcomes information. Vessels with AI-QCT-derived stenosis <20% were initially classified as nonischemic, whereas those with stenosis >80% were considered ischemic. For vessels falling within the intermediate stenosis range (20%-80%), a machine learning–based predictive model incorporating 37 quantitative features from the AI-QCT algorithm was employed to estimate the likelihood of functionally significant stenosis. A random forest algorithm achieved the highest internal performance in the derivation cohort and was selected as the final model. The final random forest model was constructed with hyperparameter tuning achieved via 10 repeated stratified 5-fold cross-validation. Bayesian hyperparameter optimization was employed to maximize the average performance over the test sets across all random splits and folds. The optimized model comprised over 1000 decision trees, each with a maximal depth of 7 layers.^7^

The diagnostic threshold for AI-QCT_ISCHEMIA_ was selected to optimize the balance between sensitivity and specificity, with a specificity target of >0.80 at the vessel territory level. A probability cutoff value of 0.31 was established, with values at or above this threshold considered abnormal. The AI-QCT_ISCHEMIA_ model has been tested and externally validated, with its diagnostic performance benchmarked against both invasive and noninvasive modalities in prior studies.^7^^,^^15^

### ICA and FFR

Patients underwent diagnostic ICA by board-certified interventional cardiologists in accordance with clinical indications and imaging standards. In the CREDENCE study, coronary arteries and side branches with a diameter ≥2.0 mm and luminal stenosis between 40% and 90% underwent FFR measurements using intracoronary or intravenous adenosine to induce hyperemia. For lesions with less than 40% stenosis, FFR was performed if deemed necessary. The images were then analyzed by an independent, blinded core laboratory for performance of quantitative coronary angiography and FFR reliability. In the PACIFIC-1 study, ICA was performed according to a standardized protocol, including dual orthogonal views for each coronary artery segment. All major coronary arteries were routinely evaluated with FFR, even in patients without visual stenoses, except for occluded or subtotal lesions (≥90%). Furthermore, intracoronary (150 μg) or intravenous (140 μg/kg/min) adenosine infusion was used to induce maximal coronary hyperemia. Angiographic images and FFR data underwent thorough review by experienced cardiologists, blinded to the noninvasive imaging results. FFR was calculated as the ratio of mean distal intracoronary pressure to mean aortic pressure, with ischemia defined by an FFR value ≤0.8.^11^^,^^16^

### MPI acquisition and interpretation

MPI in the CREDENCE study was conducted using either SPECT, PET, or cardiac magnetic resonance (CMR).^7^^,^^10^^,^^16^ In CREDENCE, interpretation of SPECT and PET images followed the 17-segment model recommended by the American Heart Association (AHA) and American College of Cardiology (ACC), and summed stress scores were computed per coronary artery territory. CMR perfusion imaging was assessed at rest and during stress phases using 16 of the 17 AHA/ACC segments (excluding the apex), with each segment classified as normal (score 0) or abnormal (score 1). These segmental scores were then summed by vascular territory, with an summed stress scores ≥1, denoting abnormal perfusion. In the PACIFIC-1 study,^11^ patients underwent PET with 370 Mbq of [^15^O]H_2_O at rest and during adenosine-induced hyperemia, with hyperemic myocardial blood flow quantified for all 3 major coronary territories; a value of ≤2.30 mL/min/g was considered abnormal. In addition, patients underwent SPECT using a 2-day stress-rest protocol, and summed difference scores were derived from the 17-segment AHA/ACC model to account for vessel territory, with summed difference scores ≥2 defined as abnormal.

### Statistical analysis

Baseline characteristics were summarized using descriptive statistics, with continuous variables presented as mean ± SD and categorical variables as frequency and percentage. Group comparisons were conducted using Student *t* test for continuous variables and the χ^2^ test for categorical variables. Subgroup analyses stratified by age and sex were performed to assess diagnostic performance at the per-vessel level. Performance metrics of AI-QCT_ISCHEMIA_, including sensitivity, specificity, positive predictive value (PPV), negative predictive value (NPV), and accuracy, were evaluated against invasive FFR (≤0.80) as the reference standard, with additional comparisons made to MPI. Receiver operating characteristic curve analysis was conducted using continuous output values, and the area under the curve (AUC) for each modality was compared with that of AI-QCT_ISCHEMIA_ using the DeLong test. All statistical analyses were performed using SPSS Statistics version 29.0.0.0 (IBM Corp) and R Studio version 4.5.0. A *P* value < .05 was considered statistically significant.

## Results

### Ischemia detection in men and women

In the CREDENCE study, women demonstrated significantly lower total plaque volume compared with men (164.28 ± 147.86 mm^3^ vs 227.64 ± 211.09 mm^3^; *P* < .001), as well as lower overall percent atheroma volume (16.33% ± 11.14% vs 18.26% ± 11.82%; *P* < .05). In addition, women had significantly less necrotic core volume (1.33 ± 2.16 mm^3^ vs 1.97 ± 3.86 mm^3^; *P* = .002), noncalcified plaque volume (104.71 ± 87.65 mm^3^ vs 151.25 ± 137.76 mm^3^; *P* < .001), and calcified plaque volume (106.04 ± 88.84 mm^3^ vs 153.22 ± 139.81 mm^3^; *P* = .02) compared with men. Women in CREDENCE were also less likely to have obstructive CAD. Similarly, findings from the PACIFIC-1 study revealed that women had significantly lower plaque volume (51.02 ± 81.07 mm^3^ vs 120.08 ± 151.65 mm^3^; *P* < .001) and plaque burden (8.99% ± 12.51% vs 17.39% ± 16.68%; *P* < .001) compared with men. Detailed characteristics are shown in Table 1.

**Table 1:** Per-vessel baseline characteristics by sex in CREDENCE and PACIFIC-1 studies.

Characteristics	CREDENCE			PACIFIC-1		
	Women ( 273 vessels)	Men (595 vessels)	*P* value	Women (226 vessels)	Men (386 vessels)	*P* value
Total plaque volume, mm^3^	164.28 ± 147.86	227.64 ± 211.09	<.001	51.02 ± 81.07	120.08 ± 151.65	<.001
Low density plaque volume, mm^3^	1.33 ± 2.16	1.97 ± 3.86	.002	0.13 ± 0.069	1.73 ± 7.28	<.001
Noncalcified plaque volume, mm^3^	104.71 ± 87.65	151.25 ± 137.76	<.001	23.28 ± 34.52	65.04 ± 80.16	<.001
Calcified plaque volume, mm^3^	106.04 ± 88.84	153.22 ± 139.81	.021	27.61 ± 53.98	53.31 ± 87.26	<.001
PAV (total), %	16.33 ± 11.14	18.26 ± 11.82	.021	8.99 ± 12.51	17.39 ± 16.68	<.001
PAV CP, %	5.60 ± 7.08	6.01 ± 7.67	.453	4.89 ± 8.82	7.70 ± 10.35	<.001
PAV NCP, %	10.59 ± 6.63	12.09 ± 7.37	.004	4.07 ± 5.07	9.47 ± 8.57	<.001
Average lumen area, mm^2^	3.98 ± 1.21	4.17 ± 1.40	.038	4.09 ± 1.74	4.62 ± 2.25	.001
Diameter stenosis, %	35.55 ± 24.19	40.57 ± 24.44	.005	17.85 ± 22.32	34.21 ± 30.31	<.001
0%	7 (2.6%)	9 (1.5%)	.009	59 (26.1%)	49 (12.7%)	<.001
1%-24%	97 (35.5%)	175 (29.4%)		105 (46.5%)	142 (36.8%)	
25%-49%	104 (38.1%)	210 (35.3%)		33 (14.6%)	63 (16.3%)	
50%-69%	32 (11.7%)	110 (18.5%)		15 (6.6%)	59 (15.3%)	
70%-99%	21 (7.7%)	61 (10.3%)		12 (5.3%)	57 (14.8%)	
100%	12 (4.4%)	30 (5.0%)		2 (0.9%)	16 (4.1%)	
Prevalence of ischemia (FFR ≤0.80)	67 (24.5%)	162 (27.2%)	.405	21 (9.3%)	144 (37.3%)	<.001

Values are mean ± SD or n (%).

CP, calcified plaque; FFR, fractional flow reserve; NCP, noncalcified plaque; PAV, percent atheroma volume.

**Figure 1:**
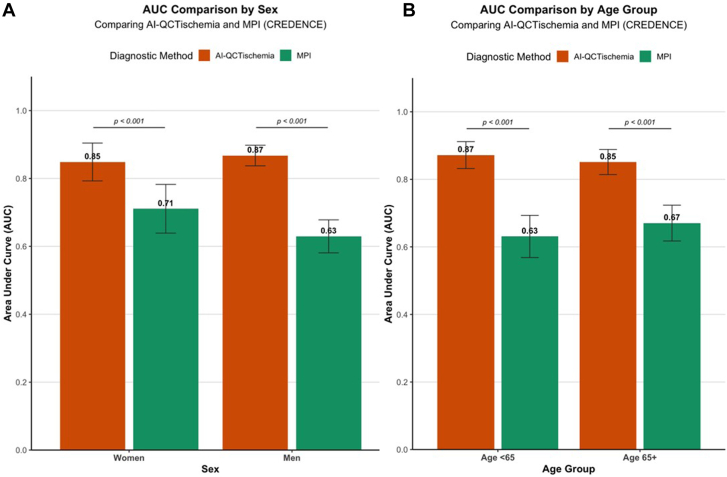
**Diagnostic Performance of AI-QCT_ISCHEMIA_ by Sex and Age group in CREDENCE.** (A) Comparison of AUCs for AI-QCT_ISCHEMIA_ (orange) and MPI (green) in women and men. (B) Comparison of AUCs for AI-QCT_ISCHEMIA_ and MPI in patients aged <65 years and ≥65 years. AI-QCT_ISCHEMIA_ showed significantly improved diagnostic performance compared with MPI in all subgroups (*P* < .001). AUC, area under the curve; MPI, myocardial perfusion imaging.

**Figure 2:**
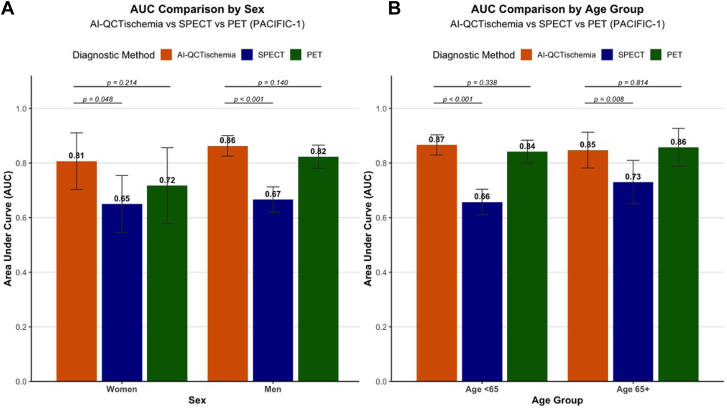
**Diagnostic performance of AI-QCT_ISCHEMIA_ by Sex and Age groups in PACIFIC-1.** (A) Comparison of AUCs for AI-QCT_ISCHEMIA_ (orange), SPECT (blue), and PET (green) in women and men from the PACIFIC-1 study population. (B) Comparison of AUCs for AI-QCT_ISCHEMIA_ (orange), SPECT (blue), and PET (green) in patients aged <65 years and ≥65 years from the PACIFIC-1 study population. AI-QCT_ISCHEMIA_ achieved significantly higher diagnostic performance than SPECT across all subgroups (*P* < .05) and demonstrated comparable performance to PET. AUC, area under the curve; PET, positron emission tomography; SPECT, single-photon emission computed tomography.

In the CREDENCE study, per-vessel analysis in women demonstrated that AI-QCT_ISCHEMIA_ achieved an AUC of 0.85 (95% CI, 0.79-0.90), which was significantly higher than MPI (AUC = 0.71; 95% CI, 0.63-0.79; *P* < .001). A similar trend was observed in men, where AI-QCT_ISCHEMIA_ yielded an AUC of 0.87 (95% CI, 0.84-0.90), outperforming MPI (AUC = 0.63; 95% CI, 0.58-0.68; *P* < .001; Figure 1A). External validation in the PACIFIC-1 cohort confirmed the consistency of these findings. In women, per-vessel analysis demonstrated that AI-QCT_ISCHEMIA_ maintained superior diagnostic performance compared with SPECT (AUC = 0.81; 95% CI, 0.70-0.92 vs AUC = 0.65, 95% CI, 0.55-0.75; *P* < .001), whereas showing comparable performance with PET (AUC = 0.72; 95% CI, 0.58-0.86; *P* = .214). A similar pattern was observed in men, where AI-QCT_ISCHEMIA_ achieved an AUC of 0.86 (95% CI, 0.83-0.90), superior to SPECT (AUC = 0.67; 95% CI, 0.61-0.73; *P* < .001) and comparable with PET (AUC = 0.82; 95% CI, 0.78-0.87; *P* = .140) (Figure 2A). The detailed diagnostic performance metrics (sensitivity, specificity, NPV, and PPV) are summarized in Table 3 and Table 4.

### Ischemia detection in older versus younger patients

In the CREDENCE cohort, patients aged ≥65 years exhibited significantly greater calcified plaque volume compared with their younger counterparts (279.09 ± 310.10 mm^3^ vs 149.26 ± 194.96 mm^3^, *P* < .001), as well as a higher total plaque volume (223.06 ± 213.35 mm^3^, *P* = .01) and overall plaque burden (19.48% ± 12.48% vs 15.64% ± 10.28%, *P* < .001). Although younger individuals showed a trend toward higher noncalcified plaque volume, the difference was not statistically significant (140.59 ± 130.34 mm^3^ vs 133.02 ± 122.00 mm^3^, *P* = .38). A similar pattern was observed in the PACIFIC-1 cohort, where older patients (≥65 years) had significantly greater total plaque volume (123.30 ± 146.50 mm^3^ vs 85.42 ± 128.90 mm^3^, *P* < .001) and plaque burden (18.52% ± 17.87% vs 12.94% ± 14.84%, *P* < .001). Calcified plaque volume was also significantly higher in the older group (69.08 ± 101.12 mm^3^ vs 35.76 ± 66.60 mm^3^, *P* < .001). No significant difference was observed in noncalcified plaque volume between age groups (53.46 ± 56.20 mm^3^ vs 48.40 ± 78.33 mm^3^, *P* = .44). Detailed characteristics are summarized in Table 2.

**Table 2:** Per-vessel baseline characteristics by age groups in CREDENCE and PACIFIC-1 studies.

Characteristics	CREDENCE			PACIFIC-1		
	Age <65 years (412 vessels)	Age ≥65 years (456 vessels)	*P* value	Age <65 years (464 vessels)	Age ≥65 years (148 vessels)	*P* value
Total plaque volume, mm^3^	190.72 ± 172.50	223.06 ± 213.35	.014	85.42 ± 128.93	123.30 ± 146.50	.001
Low density plaque volume, mm^3^	2.14 ± 4.29	1.44 ± 2.36	.003	1.26 ± 6.50	0.76 ± 2.98	.366
Noncalcified plaque volume, mm^3^	140.59 ± 130.34	133.02 ± 122.0	.377	48.40 ± 78.33	53.46 ± 56.20	.444
Calcified plaque volume, mm^3^	47.99 ± 75.82	88.61 ± 124.27	<.001	35.76 ± 66.60	69.08 ± 101.12	<.001
PAV (total), %	15.64 ± 10.28	19.48 ± 12.48	<.001	12.94 ± 14.84	18.52 ± 17.87	<.001
PAV CP, %	3.99 ± 5.74	7.60 ± 8.43	<.001	5.50 ± 8.48	10.30 ± 12.77	<.001
PAV NCP, %	11.47 ± 7.28	11.75 ± 7.09	.576	7.27 ± 8.16	8.13 ± 7.04	.217
Average lumen area, mm^2^	4.28 ± 1.37	3.95 ± 1.29	<.001	4.48 ± 2.05	4.24 ± 2.21	.225
Diameter stenosis, %	37.30 ± 24.19	40.53 ± 24.63	.052	25.97 ± 28.02	35.06 ± 29.86	<.001
0%	8 (1.9%)	8 (1.8%)	.179	95 (20.5%)	13 (8.8%)	<.001
1%-24%	143 (34.7%)	129 (28.3%)		192 (41.4%)	55 (37.2%)	
25%-49%	148 (35.9%)	166 (36.4%)		65 (14%)	31 (20.9%)	
50%-69%	55 (13.3%)	87 (19.1%)		54 (11.6%)	20 (13.5%)	
70%-99%	41 (10%)	41 (9.0%)		48 (10.3%)	21 (14.2%)	
100%	17 (4.1%)	25 (5.5%)		10 (2.2%)	8 (5.4%)	
Prevalence of ischemia (FFR ≤0.80)	100 (24.3%)	129 (28.3%)	.063	120 (25.9%)	45 (30.4%)	.292

Values are mean ± SD or n (%).

CP, calcified plaque; FFR, fractional flow reserve; NCP, noncalcified plaque; PAV, percent atheroma volume.

AI-QCT_ISCHEMIA_ demonstrated consistent performance across age groups. In the CREDENCE cohort, per-vessel analysis showed that in the older population, AI-QCT_ISCHEMIA_ achieved an AUC of 0.85 (95% CI, 0.81-0.89), significantly higher than MPI (AUC = 0.67; 95% CI, 0.61-0.73; *P* < .001). Similarly, in the younger population, AI-QCT_ISCHEMIA_ had an AUC of 0.87 (95% CI, 0.83-0.91), surpassing MPI (AUC = 0.63; 95% CI, 0.56-0.70; *P* < .001) (Figure 1B). In the PACIFIC-1 study, among older individuals, AI-QCT_ISCHEMIA_ showed an AUC of 0.85 (95% CI, 0.78-0.91), significantly higher than SPECT (AUC = 0.73; 95% CI, 0.63-0.83; *P* = .008) and comparable with PET (AUC = 0.86; 95% CI, 0.79-0.93; *P* = .814). In the younger cohort, AI-QCT_ISCHEMIA_ had an AUC of 0.87 (95% CI, 0.83-0.91), outperforming SPECT (AUC = 0.66; 95% CI, 0.60-0.72; *P* < .001) and showing similar performance to PET (AUC = 0.84; 95% CI, 0.80-0.88; *P* = .338) (Figure 2B). Details on sensitivity, specificity, NPV, and PPV are provided in Tables 3 and 4.

**Table 3:** Diagnostic metrics of AI-QCT_ISCHEMIA_ compared with MPI in the CREDENCE study population.

Test	Sensitivity	Specificity	PPV	NPV	Accuracy
CREDENCE: women (273 vessels)					
AI-QCT_ISCHEMIA_	71.6% (48/67)	87.9% (181/206)	65.8% (48/73)	90.5% (181/200)	83.9% (229/273)
MPI	65.7% (44/67)	68.0% (140/206)	40.0% (44/110)	85.9% (140/163)	67.4% (184/273)
CREDENCE: men (595 vessels)					
AI-QCT_ISCHEMIA_	79.0% (128/162)	78.5% (340/433)	57.9% (128/221)	90.9% (340/374)	78.7% (468/595)
MPI	55.6% (90/162)	64.2% (278/433)	36.7% (90/245)	79.4% (278/350)	61.8% (369/595)
CREDENCE: ≥65 years old (456 vessels)					
AI-QCT_ISCHEMIA_	77.5% (100/129)	79.2% (259/327)	59.5% (100/168)	89.9% (259/288)	78.7% (359/456)
MPI	62.0% (82/129)	66.7% (218/267)	42.3% (80/189)	81.6% (218/267)	65.4% (298/456)
CREDENCE: <65 years old (412 vessels)					
AI-QCT_ISCHEMIA_	76.0% (76/100)	84.0% (262/312)	60.3% (76/126)	91.6% (262/286)	82.0% (338/412)
MPI	54.0% (54/100)	64.1% (200/312)	32.5% (54/166)	81.3% (200/246)	61.7% (254/412)

Values are % (n/N).

MPI, myocardial perfusion imaging.

**Table 4:** Diagnostic metrics of AI-QCT_ISCHEMIA_ compared with SPECT and PET in the PACIFIC-1 study population.

Test	Sensitivity	Specificity	PPV	NPV	Accuracy
PACIFIC-1: women (226 vessels)					
AI-QCT_ISCHEMIA_	66.7% (14/21)	88.3% (181/205)	36.8% (14/38)	96.3% (181/188)	86.3% (195/226)
SPECT	33.3% (7/21)	96.6% (198/205)	50.0% (7/14)	93.4% (198/212)	90.7% (205/226)
PET	47.6% (10/21)	91.7% (188/205)	37.0% (10/27)	94.5% (188/199)	87.6% (198/226)
PACIFIC-1: men (386 vessels)					
AI-QCT_ISCHEMIA_	77.1% (111/144)	80.6% (195/242)	70.3% (111/158)	85.5% (195/228)	79.3% (306/386)
SPECT	42.4% (61/144)	88.8%(215/242)	69.3% (61/88)	72.1% (215/298)	71.5% (276/386)
PET	86.1% (124/144)	66.9% (162/242)	60.8% (124/204)	89.0% (162/182)	74.1% (286/386)
PACIFIC-1: ≥65 years old (148 vessels)					
AI-QCT_ISCHEMIA_	80% (36/45)	76.7% (79/103)	60.0% (36/60)	89.8% (79/88)	77.7% (115/148)
SPECT	48.9% (22/45)	91.3% (94/103)	71.0% (22/31)	80.3% (94/117)	78.4% (116/148)
PET	86.7% (39/45)	72.8% (75/103)	58.2% (39/67)	92.6% (75/81)	77.0% (114/148)
PACIFIC-1: <65 years old (464 vessels)					
AI-QCT_ISCHEMIA_	74.2% (89/120)	86.3% (297/344)	65.4% (89/136)	90.5% (297/328)	83.2% (386/464)
SPECT	38.3% (46/120)	92.7% (319/344)	64.8% (46/71)	81.2% (319/393)	78.7% (365/464)
PET	79.2% (95/120)	79.9% (275/344)	57.9% (95/164)	91.7% (275/300)	79.7% (370/464)

Values are % (n/N).

PET, positron emission tomography; SPECT, single-photon emission computed tomography.

## Discussion

In this study, we evaluated the diagnostic performance of a novel AI-guided quantitative coronary computed tomography algorithm, AI-QCT_ISCHEMIA_, for predicting myocardial ischemia across key demographic subgroups. The principal finding is that the algorithm demonstrated consistently high diagnostic accuracy in both men and women, as well as in younger and older patients, with improved performance compared with standard nuclear imaging techniques such as MPI (Central Illustration). The robustness of AI-QCT_ISCHEMIA_ across 2 independent cohorts, despite notable baseline differences in atherosclerotic plaque characteristics among these subgroups, supports its potential utility as a reliable noninvasive tool for diagnosing hemodynamically significant CAD.

**Central Illustration:**
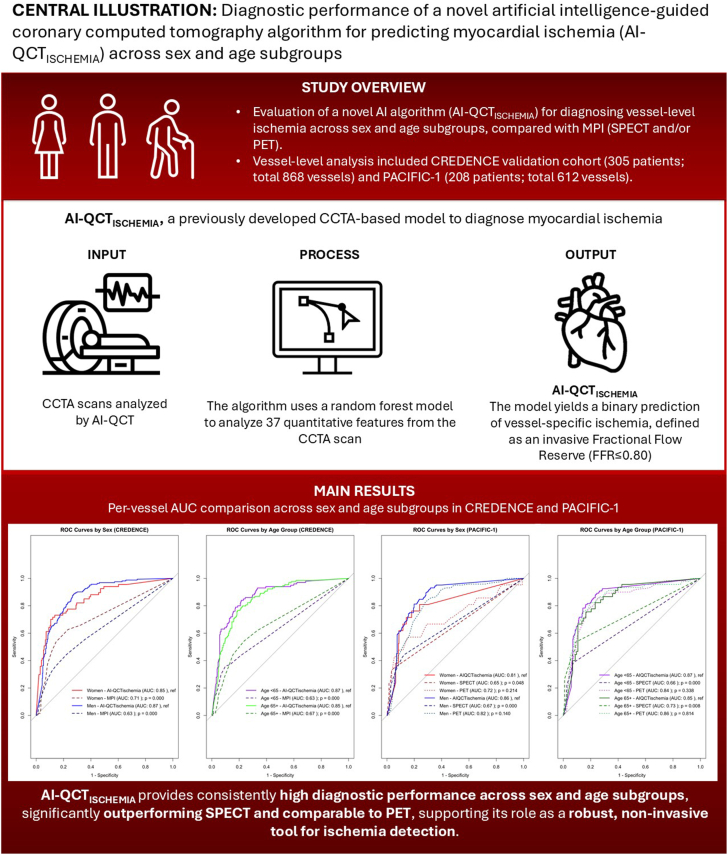
**Diagnostic performance of AI-QCT**_**ISCHEMIA**_**for vessel-specificcoronary ischemia across sex and age subgroups.** Receiver operating characteristic curve analyses demonstrated consistently high diagnostic performance of AI-QCT_ISCHEMIA_ across sex and age subgroups, outperforming SPECT and showing comparable performance to PET. These findings highlight the potential AI-QCT_ISCHEMIA_ as a reliable noninvasive approach for ischemia detection.

### Diagnostic performance of AI-QCT_ISCHEMIA_ in men versus women

Consistent with prior literature,^2^^,^^17^ men in our study exhibited a higher prevalence of ischemia, lower FFR values, and greater plaque burden compared with women. These findings emphasize the importance of incorporating sex-specific considerations when evaluating diagnostic tools, particularly for women, who often present with unique disease manifestations. Unlike men, women tend to have a higher prevalence of nonobstructive stenosis and a tendency to present at an older age and/or with atypical symptoms.^18^^,^^19^ However, traditional diagnostic modalities are often suboptimal in women due to anatomical and physiological differences.^17^ These challenges can result in delayed or missed diagnoses, although women with ischemia have similar or even greater risks of adverse outcomes and diminished quality of life as compared with men.^20^ This diagnostic gap highlights a critical unmet need for more tailored noninvasive strategies that maintain accuracy across sexes.

In our study, AI-QCT_ISCHEMIA_ demonstrated high diagnostic accuracy in both sexes across both cohorts, compared with SPECT. Although MPI remain valuable noninvasive tools, its diagnostic performance may be influenced by sex-specific anatomical and physiological factors.^21^^,^^22^ For instance, SPECT imaging has limitations in women due to the smaller heart size, which potentially reduces sensitivity for detecting obstructive CAD; also, breast attenuation may result in false-positive SPECT results.^23^^,^^24^ In addition, AI-QCT_ISCHEMIA_ performed comparably to PET in both sexes, supporting its role as a balanced diagnostic tool across sex-specific disease profiles.

Although AI-QCT_ISCHEMIA_ provides reliable diagnostic performance in both men and women, subtle sex-specific differences were observed. In women, the algorithm yielded slightly lower sensitivity but higher specificity and notably high NPV, making it a particularly valuable rule-out tool in clinical settings where diagnostic uncertainty is common. In men, the model exhibited slightly higher sensitivity with comparable specificity, underscoring its effectiveness in detecting ischemia, which is more prevalent in this group. Importantly, these subtle variations did not result in a statistically significant difference in overall diagnostic performance, confirming the algorithm's strong and balanced performance profile across both sexes.

The diagnostic strength of AI-QCT_ISCHEMIA_ aligns with prior studies, suggesting that AI-based diagnostic tools provide improved accuracy compared with traditional methods such as MPI.^15^^,^^25^^,^^26^ The model’s high performance likely stems from its multiparametric analytical framework, which integrates 37 AI-derived quantitative parameters, including stenosis severity, plaque characteristics, and vascular morphology,^7^ enabling precise ischemia detection. This integrative approach suggests that AI-QCT_ISCHEMIA_ may serve as a reliable, sex-inclusive alternative for myocardial ischemia assessment.

### Diagnostic performance of AI-QCT_ISCHEMIA_ across different age groups

The current study also highlighted distinct age-related patterns in coronary artery pathology: older patients demonstrated higher ischemia prevalence, greater plaque burden, and more calcified plaques, whereas younger individuals showed predominance of noncalcified plaques, aligning with previous findings.^27^, ^28^, ^29^, ^30^ Despite these differences, AI-QCT_ISCHEMIA_ maintained high diagnostic performance across all age groups, significantly outperforming SPECT and showing comparable performance with PET.

The superior performance of AI-QCT_ISCHEMIA_ in older adults is particularly noteworthy, given the high prevalence and complexity of CAD in the elderly, where precise diagnostic tools are critical for effective clinical management.^31^ Importantly, its diagnostic strength also extended to younger patients, who typically present with lower disease prevalence and different clinical profiles. Accurate ischemia detection in younger individuals is clinically important, as it enables timely identification of functionally significant coronary disease and may inform early therapeutic decisions that prevent progression and adverse cardiac events. These observations support the potential applicability of AI-QCT_ISCHEMIA_ as a noninvasive diagnostic method suitable across a broad age spectrum.

The improved diagnostic performance of AI-QCT_ISCHEMIA_ relative to conventional modalities stems from its machine learning architecture, which mitigates some limitations of existing noninvasive ischemia testing.^15^^,^^25^^,^^32^ Moreover, it may offer advantages in situations where conventional MPI is less informative due to technical or patient-specific limitations.^33^ In PACIFIC-1, [^15^O]H_2_O PET was used, regarded as the ‘‘gold standard’’ method for absolute measurement of myocardial blood flow, although its broader clinical use is constrained by limited availability.^34^^,^^35^ The comparable performance of AI-QCT_I__SCHEMIA_ against PET in this study supports its potential role as a practical and scalable alternative, particularly where PET is not routinely accessible. These findings suggest that AI-QCT_ISCHEMIA_ may complement existing modalities by enhancing diagnostic consistency and reliability across a broad range of anatomical and clinical scenarios.

### Clinical Integration of AI-QCT_ISCHEMIA_

The consistent diagnostic accuracy of AI-QCT_ISCHEMIA_ across sex and age subgroups supports its potential for integration into routine clinical workflows. Using quantitative parameters obtainable from standard CCTA, the algorithm may help reduce diagnostic uncertainty, minimize the need for additional functional testing, and broaden the role of CCTA beyond purely anatomical assessment. Our findings show that an AI-guided CCTA algorithm can predict functionally significant ischemia directly from anatomical features, making it a comprehensive tool that combines the assessment of stenosis, plaque burden, and ischemia in a single test. Refinement at the coronary segment level could further increase its clinical value, particularly in revascularization planning.

### Limitations

This study has several limitations. Since this is a post-hoc analysis of 2 multicenter trials, there is a potential for selection bias, which may affect the generalizability of the findings to wider patient populations. Moreover, although combining data from the CREDENCE and PACIFIC-1 cohorts enhanced the sample size and diversity, inherent differences in study design, patient characteristics, and imaging protocols may have introduced some variability in the results. Ischemia prediction was evaluated at the vessel level, whereas segment level assessment (eg, proximal vs distal segments) was beyond the scope of this study but may provide additional procedural insights and warrants investigation in future studies. Finally, although this study focused on diagnostic accuracy, future investigations should explore clinical outcomes, cost-effectiveness, and workflow integration to better define its role in practice.

## Conclusion

The AI-QCT_ISCHEMIA_ demonstrates high diagnostic performance for the detection of myocardial ischemia, significantly outperforming standard nuclear perfusion imaging, such as SPECT, and showing comparable performance with PET across sex and age subgroups. By leveraging comprehensive AI-driven quantitative parameters, AI-QCT_ISCHEMIA_ offers a more precise assessment across various patient demographic characteristics. These findings suggest that AI-QCT_ISCHEMIA_ may contribute to reducing diagnostic uncertainty and enhancing timely, informed clinical decision making across a broad range of patients. Future prospective studies are warranted to evaluate its role in guiding therapy and improving clinical outcomes.

## Declaration of competing interest

Gianluca Pontone received an honorarium as a speaker/consultant and an institutional research grant from GE Healthcare, Bracco, Medtronic, and Novartis. Ibrahim Danad is a member of the Cleerly Scientific Advisory Board. Nick S. Nurmohamed reports grants from the Dutch Heart Foundation (Dekker 03-007-2023-0068), European Atherosclerosis Society (2023), research funding/speaker fees from Cleerly, Daiichi Sankyo, and Novartis, and is co-founder of Lipid Tools. Alexander van Rosendael is a member of the Cleerly Scientific Advisory Board. The remaining authors have no conflicts of interest to declare.

## The CREDENCE and PACIFIC-1 investigators

Ran Heo, MD, PhD¹, Hyung-Bok Park, MD^2^, Hugo Marques, MD, PhD^3^, Wijnand J. Stuijfzand, MD, PhD⁴, Jung Hyun Choi, MD, PhD^5^, Joon-Hyung Doh, MD^6^, Ae-Young Her, MD, PhD^7^, Bon-Kwon Koo, MD, PhD^8^, Chang-Wook Nam, MD, PhD^9^, Sang-Hoon Shin, MD^10^, Jason Cole, MD^11^, Alessia Gimelli, MD^12^, Muhammad Akram Khan, MD^13^, Bin Lu, MD^14^, Yang Gao, MD^14^, Faisal Nabi, MD^15^, Mouaz H. Al-Mallah, MD^15^, Ryo Nakazato, MD^16^, U. Joseph Schoepf, MD^17^, Randall C. Thompson, MD^18^, James J. Jang, MD^19^, Michael Ridner, MD^20^, Chris Rowan, MD^21^, Erick Avelar, MD^22^, Philippe Généreux, MD^23^, Guus A. de Waard, MD^4^

^1^Division of Cardiology, Department of Internal Medicine, Hanyang University Medical Center, Seoul, South Korea; ^2^Division of Cardiology, Department of Internal Medicine, International St. Mary's Hospital, Catholic Kwandong University College of Medicine, Incheon, South Korea; ^3^Faculdade de Medicina da Universidade Católica Portuguesa, Lisboa, Portugal; ^4^Department of Cardiology, Amsterdam UMC, Vrije Universiteit Amsterdam, Amsterdam, the Netherlands; ^5^Department of Cardiology, Pusan National University Hospital, Busan, South Korea; ^6^Division of Cardiology, Inje University Ilsan Paik Hospital, South Korea; ^7^Division of Cardiology, Department of Internal Medicine, Kangwon National University, College of Medicine, Kangwon National University School of Medicine, Chuncheon, South Korea; ^8^Department of Internal Medicine, Seoul National University Hospital, Seoul, South Korea; ^9^Cardiovascular Center, Keimyung University Dongsan Hospital, Daegu, South Korea; ^10^Division of Cardiology, Department of Internal Medicine, Ewha Women's University Seoul Hospital, Seoul, South Korea; ^11^Mobile Cardiology Associates, Mobile, Alabama; ^12^Department of Imaging, Fondazione Toscana Gabriele Monasterio, Pisa, Italy; ^13^Cardiac Center of Texas, McKinney, Texas; ^14^State Key Laboratory of Cardiovascular Disease, Fuwai Hospital, Beijing, China; ^15^Houston Methodist Hospital, Houston, Texas; ^16^Cardiovascular Center, St. Luke's International Hospital, Tokyo, Japan; ^17^Medical University of South Carolina, Charleston, South Carolina; ^18^Saint Luke's Mid America Heart Institute, Kansas City, Missouri; ^19^San Jose Medical Center, Kaiser Permanente, San Jose, California; ^20^University of Alabama-Birmingham, Birmingham, Alabama; ^21^University of Wisconsin-Madison, Madison, Wisconsin; ^22^Oconee Heart and Vascular Center, University of Georgia School of Medicine, Athens, Georgia; ^23^Morristown Medical Center, Morristown, New Jersey
